# Effect of systemic antibiotics on the outcomes of regenerative periodontal surgery in intrabony defects: a randomized, controlled, clinical study

**DOI:** 10.1007/s00784-020-03616-7

**Published:** 2020-10-13

**Authors:** Małgorzata Pietruska, Ewa Dolińska, Robert Milewski, Anton Sculean

**Affiliations:** 1 Department of Periodontal and Oral Mucosa Diseases, Medical University of Bialystok, ul.Waszyngtona 13, 15-269 Bialystok, Poland; 2 Department of Statistics and Medical Informatics, Medical University of Bialystok, ul.Szpitalna 37, 15-295 Bialystok, Poland; 3 Department of Periodontology, School of Dental Medicine, University of Bern, Freiburgstrasse 7, 3010 Bern, Switzerland

**Keywords:** Periodontal intrabony defects, Periodontal regeneration, Guided tissue regeneration, Deproteinized bovine bone mineral, Systemic antibiotics

## Abstract

**Objectives:**

To assess the potential influence of systemic antibiotic administration on the healing of periodontal intrabony defects treated with deproteinized bovine bone mineral (DBBM) and collagen membrane.

**Materials and methods:**

Forty-one intrabony defects were treated by means of DBBM and collagen membrane (GTR). Postoperatively, the patients received either systemic antibiotics (i.e., 1 g of amoxicillin, twice daily for 7 days) (test) or no antibiotics (control). Clinical attachment level (CAL), probing depth (PD), and gingival recession (GR) were measured at baseline and at 1 year following regenerative surgery. The depth of the intrabony component (INTRA DD) and its width (INTRA DW) were measured during surgery and after 1 year at reentry. The depth (RxD) and width (RxW) of the intrabony defects were evaluated radiographically at baseline and at 1 year.

**Results:**

No adverse events were observed in any of the two groups throughout the entire study period. In the test group, mean CAL changed from 8.7 ± 1.4 mm at baseline to 5.0 ± 1.7 mm at 1 year (*p* < 0.0001), while PD decreased from 7.8 ± 1.5 mm at baseline to 4.0 ± 0.9 mm at 1 year (*p* < 0.0001). In the control group, mean CAL changed from 8.6 ± 1.9 mm to 5.9 ± 1.6 mm (*p* < 0.001) and mean PD improved from 7.4 ± 1.3 mm to 4.1 ± 1.3 mm (*p* < 0.001). Mean CAL gain measured 3.6 ± 1.6 mm in the test and 2.7 ± 1.6 mm in the control group, respectively. Defect fill (i.e., INTRA DD gain) at re-entry measured 3.7 ± 1.8 mm in the test and 2.7 ± 2.1 mm in the control group. A CAL gain of ≥ 3 mm was measured in 76% of the defects in the test group and in 40% of the defects in the control group, respectively. In both groups, all evaluated clinical and radiographic parameters improved statistically significantly compared with baseline, but no statistically significant differences were found between the two groups.

**Conclusions:**

Within their limits, the present study has failed to show any substantial added clinical benefits following the postoperative administration of amoxicillin in conjunction with regenerative periodontal surgery using DBBM and GTR.

**Clinical relevance:**

The post-surgically administration of systemic antibiotics does not seem to be necessary following regenerative periodontal surgery.

## Introduction

The goal of regenerative periodontal surgery is to reconstruct the tooth’s supporting tissues (i.e., periodontal ligament, root cementum, and bone) that have been lost following inflammatory periodontal disease or trauma [1, 2]. A plethora of different treatment modalities including tissue preservation flaps in conjunction with bone grafts/bone substitutes, guided tissue regeneration (GTR), enamel matrix derivative (EMD), growth factors, or various combination thereof, have been widely employed in order to facilitate periodontal regeneration in intrabony and furcation defects [1–5]. Substantial evidence from long-term clinical studies indicates that regenerative periodontal surgery represents a realistic treatment modality for improving the prognosis of periodontally compromised teeth, thus contributing to tooth maintenance [6–9]. The combination of a deproteinized bovine bone mineral (DBBM) and collagen membrane (GTR) is a widely used and well-documented treatment modality in regenerative periodontal surgery [1, 10–12].

In most clinical scenarios, systemic antibiotics are routinely given following regenerative periodontal surgery to reduce postoperative complications caused by bacterial infections. However, the data from controlled clinical studies evaluating the potential influence of a postoperative administration of systemic antibiotics following regenerative periodontal surgery is still limited [13, 14]. However, the use of regenerative biomaterials such as bone grafts and membranes may increase the risk of postoperative complications such as membrane exposure and subsequent bacterial colonization, thus jeopardizing the clinical outcomes. Therefore, in order to minimize these potential complications, systemic antibiotics are frequently administered after regenerative periodontal surgery involving the use of bone grafts and barrier membranes [15]. However, at present, there are virtually no data from randomized controlled clinical studies evaluating the use of systemic antibiotics following regenerative periodontal surgery by means of DBBM and GTR.

Therefore, the aim of the present study was to evaluate the potential effect of systemic antibiotics administration following regenerative periodontal surgery with DBBM and GTR.

## Material and methods

### Study sample and experimental design

The study was planned as a randomized, prospective, controlled clinical trial with reentry procedures. Prior to patient recruitment, the study protocol was approved by the local ethical committee (R-I-002-302-2013) in accordance with the Helsinki Declaration of 1975 as revised in 2000. Following screening and detailed explanation about the aim and scope of the study, all patients participating in the study have signed the inform consent. The informed consent contained also detailed information about the planned re-entry procedures at the 12-month evaluation period and could freely agree or not with the possibility of a re-entry surgery.

A total of 41 patients diagnosed with stage III periodontitis [16] and having at least one intrabony defect that were referred for periodontal therapy to the Department of Periodontal and Oral Mucosa Diseases, Medical University of Bialystok were included in the study.

The criteria for inclusion were:

- Presence of at least one intrabony defect with a probing depth (PD) ≥ 6 mm associated with an intrabony component exhibiting a radiographic depth (RxD) of ≥ 3 mm and a width (RxW) of ≥ 2 mm as measured on the intraoral radiographs
- At least 18 years of age
- No allergic reaction to amoxicillin or other antibiotics belonging to the penicillin family
- Good level of oral hygiene evidenced by full mouth plaque scores (FMPS < 20%) [17], and full mouth bleeding scores (FMBS < 20%) [18]
- Non smoking

The exclusion criteria were:

- Intake of antibiotics within at least 3 months prior to the study
- Systemic diseases such as diabetes, immunodeficiencies and others that may affect wound healing
- Pregnancy and breastfeeding

**Fig. 1:**
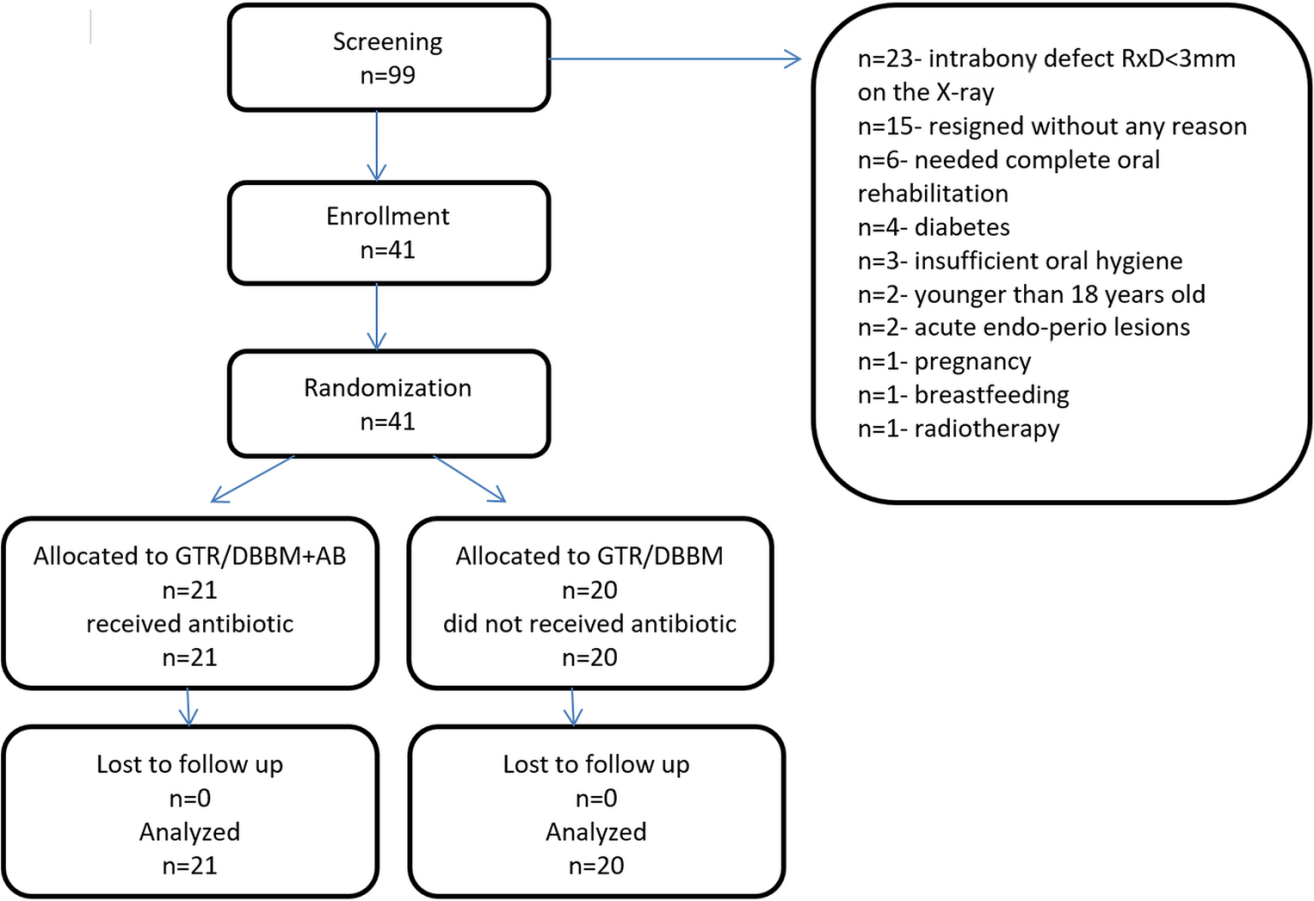
Consort flowchart of the study

The patients were distributed in the test and control groups using random allocation by means of a computer program (written for this purpose by RM). Patient’s allocation is depicted in Fig. 1.

### Surgical procedure

In order to standardize the surgical technique, the same experienced surgeon (MP) performed all interventions under local anesthesia (Ubistesin forte, 3M ESPE, Seefeld, Germany). The surgeon has only met the patient immediately before regenerative surgery and at the reentry surgery (in cases where re-entry was performed). The surgical technique consisted of intrasulcular incisions and preparation of mucoperiosteal flaps buccally and lingually according to the principles of papilla preservation technique [3, 4]. Vertical incisions were only made if needed to obtain easier access to the defect. Subsequently, the granulation tissue was removed and the root surfaces were thoroughly scaled and planed by means of hand instruments (i.e., Gracey currettes, Hu-Friedy, Chicago, IL, USA) and ultrasonic scalers (EMS Piezon Tip PS, EMS, Nyon, Switzerland).

Following site preparation, the defects were filled with the DBBM (Cerabone®, botiss biomaterials GmbH, Zossen, Germany) and covered with a collagen membrane (GTR) (Collprotect®, botiss biomaterials GmbH, Zossen, Germany). Once the membrane was in place, the mucoperiosteal flap was coronally displaced and stabilized over the defect by means of vertical modified mattress sutures, while the remaining papillae and vertical incisions were closed with single interrupted sutures (Ethilon 5.0, Johnson & Johnson Company, New Brunswick, NJ, USA).)

Following defect closure, the randomization envelope was opened and the patients were randomly distributed in the test and control group. Patients in the test group (DBBM/GTR+AB) were given postoperatively systemic antibiotics, i.e., amoxicillin (Ospamox, Sandoz GmbH, Kundl, Austria) starting the day of the surgery and followed by 2 × 1 g/day for 7 days. No antibiotics were given for the patients in the control group (DBBM/GTR). Since, in the present study, no placebo was administered, the used drug was not blinded. The antibiotics were given to the patients in their original packages. Patients were randomly allocated in the two different treatment groups. However, since no placebo was used, the patients were aware of the allocated treatment.

### Clinical and radiographic measurements

All clinical measurements were made by the same blinded (i.e., the examiner was not aware of the allocated treatments) and calibrated examiner (ED) who was not the same as the surgeon. Examiner calibration was performed as follows: five patients, not enrolled in the study, and showing at least 4 teeth with probing depths ≥ 6 mm on at least one aspect of each tooth, were evaluated by the examiner on 2 separate occasions, 48 h apart. Calibration was accepted if measurements at baseline and at 48 h were similar to the millimeter at ≥ 90%.

The following clinical parameters were measured at baseline (i.e., before surgery) and at 12 months postoperatively: probing depth (PD), gingival recession (GR), and clinical attachment level (CAL) using the same type of periodontal probe (i.e., PCPUNC 15, Hu-Friedy, Chicago, IL, USA).

Six points were probed at each tooth with the intrabony defect—mesio-, mid-, disto-buccal and mesio-, mid-, disto-lingual. The fixed reference point was cemento-enamel junction (CEJ) or a filling’s margin if CEJ was not detectable. However, in the calculations, the same, at baseline, the deepest site also exhibiting ≥ 6 mm was included.

Full mouth plaque scores (FMPS) [17] and full mouth bleeding scores (FMBS) [18] were dichotomously calculated as a percentage on the four surfaces of each tooth.

Intraoral radiographs (Planmeca Intra, serial no.: ITH13227, tube type: D-0711SB, tube no.: 27899, Planmeca, Helsinki, Finland) were taken in an analogue way at baseline and at the 12-month evaluation. The radiographs had the following settings for the different groups of teeth:

Exposition: maxillary incisors: 60 kV, 8 mA, 0.160 s/mandibular incisors: 60 kV, 8 mA, 0.125 s/maxillary premolars: 63 kV, 8 mA, 0.160 s/mandibular premolars: 63 kV, 8 mA, 0.125 s, maxillary molars: 63 kV, 8 mA, 0.2 s/mandibular molars: 63 kV, 8 mA, 0.160 s.

To ensure accuracy, a long cone parallel technique positioner was individually prepared for each of the included patients. The used film was Dental Kodak Film Carestream E-Speed Intraoral E-150 Adult Size 2 (31 mm × 41mm) (Rochester, NY, USA). The machine used to develop the films was Dürr Dental XR24 (Dürr Dental SE, Bietigheim-Bissingen, Germany). Individual intraoral film holder was made on the basis of RINN XCP® Holding System (Densply RINN, York, USA). Following manufacturing of individualized bite blocks for bite registration, a silicone impression mass was placed to the top and bottom of the bite block (O-bite, DMG, Ridgefield Park, NJ, USA). The silicone impression material was fixed to the individualized bite blocks using acrylic glue (Universal Tray Adhesive, Zhermack, Italy). Patients were biting on the bite blocks with the impression mass while the x-rays were made using the long cone parallel technique. Subsequently, the positioner was disinfected and stored. This procedure enabled to take the 1-year x-ray in the same position as at baseline. To analyze the radiographs, a negatoscope for viewing medical x-rays was used (BakMed PF-622.4, BakMed, Łódź, Poland).

The following measurements were made on the intraoral radiographs: defect depth (RxD) (i.e., the vertical distance between the alveolar bone crest and the point on the root where the width of periodontal ligament appeared to have a physiologic appearance) and defect width (RxW) (i.e., the horizontal space between the root surface and the most coronal point of the bone crest). The measurements were made at a 2.5 magnification using a millimeter grid [19, 20].

### Intrasurgical measurements

Intrasurgically the following measurements were made:

- Depth of the intrabony defect (DD) defined as the distance from the most coronal point on the alveolar bone crest to the most apical point of the intrabony defect
- Width of the intrabony defect (DW) defined as the distance from the most coronal point of the bone crest to the root [21].

**Fig. 2:**
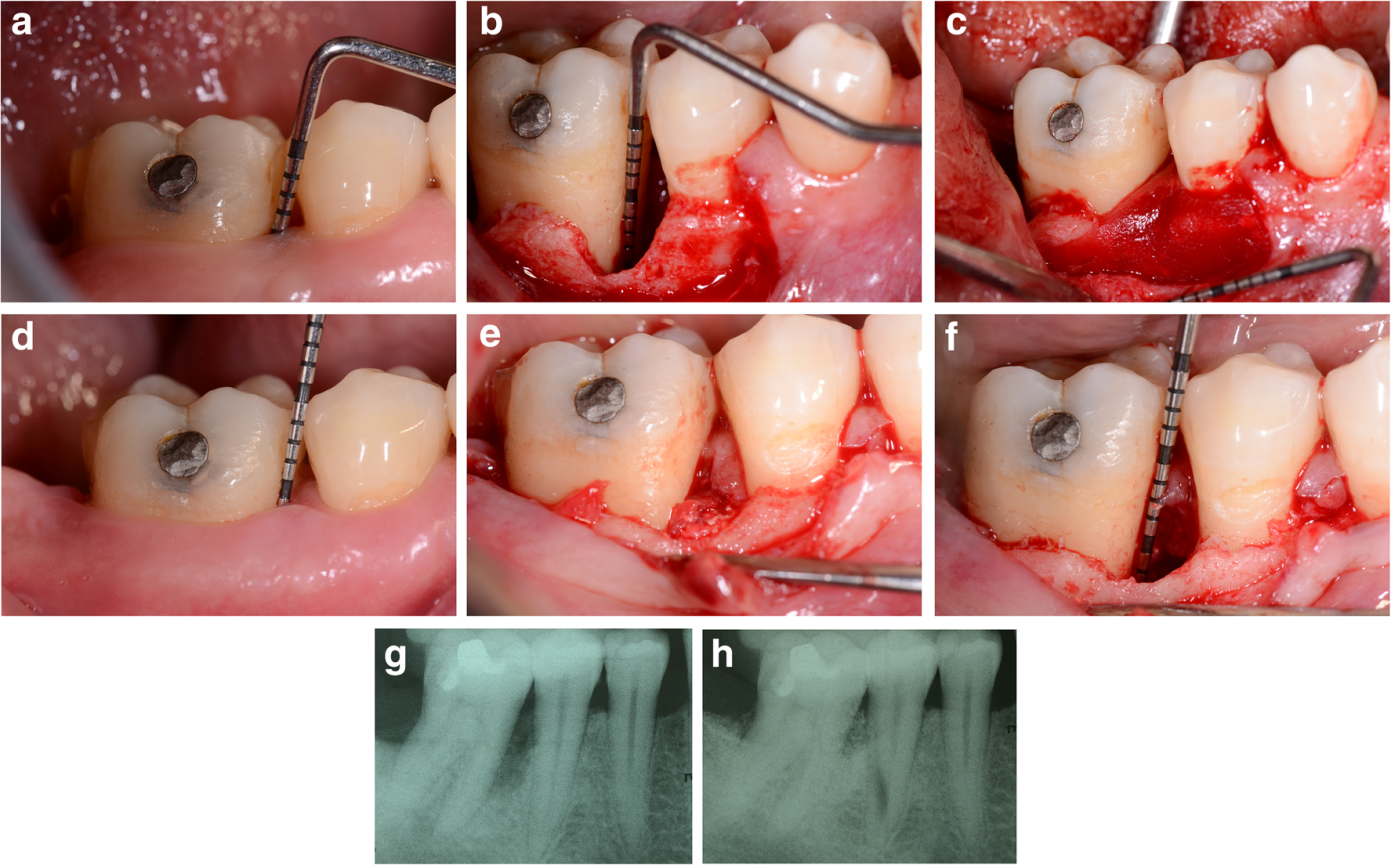
Representative bone defect treated with DBBM/GTR + AB (test group). (a) Baseline clinical view, lower right first molar (46) presenting with a pre-operative probing depth (PD) of 10 mm. (b) Surgical view after flap elevation and granulation tissue removal, intrasurgical probing of the defect (INTRA DD = 8 mm). (c) Surgical view after filling the defect with Cerabone® (botiss, biomaterials GmbH, Zossen, Germany) and covering with trimmed collagen membrane (Collprotect®, botiss, biomaterials GmbH, Zossen, Germany). (d) One year post-op clinical view, lower right first molar presenting with a post-operative probing depth (PD) of 4 mm. (e) One year post-op minimally invasive reentry; view of the buccal bone plate that has been restored. (f) Reentry; probing of the residual defect INTRA DD = 2 mm (after granulation tissue removal). (g) Baseline radiographic aspect of the intrabony defect distally to the lower right first molar. (h) Radiographic result 1 year after treatment

**Fig. 3:**
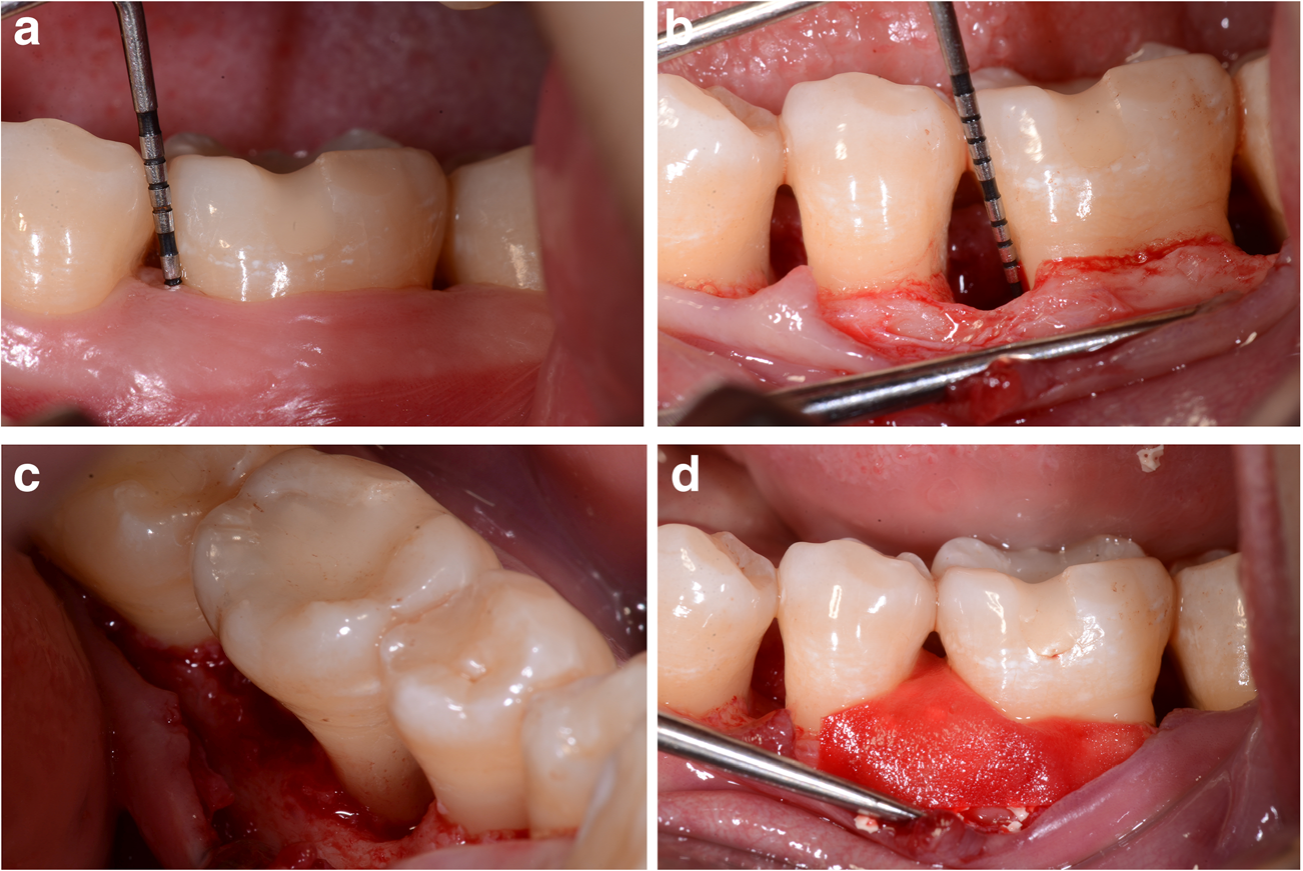
Representative bone defect treated with DBBM/GTR (control group). (a) Baseline clinical view, lower left molar (36) presenting with a pre-operative probing depth (PD) of 9 mm. (b) Surgical view after flap elevation and granulation tissue removal, presenting with a intraoperative probing of the defect INTRA DD = 5 mm. (c) Surgical view of the lingual aspect of the bony defect. (d) Intrasurgical view of the defect filled with Cerabone® (botiss, biomaterials GmbH, Zossen, Germany) and covered with trimmed collagen membrane (Collprotect®, botiss, biomaterials GmbH, Zossen, Germany). (e) Clinical view 1 year after treatment; a post-operative probing depth (PD) of 4 mm. (f, g) One year post-op minimally invasive reentry; complete filling of the defect with bonelike tissue. (h) Reentry; probing of the residual defect INTRA DD = 3 mm (after granulation tissue removal). (i) Baseline radiographic aspect of intrabony defect mesially to the lower left first molar. (j) Radiographic result 1 year after treatment

The defects were also classified according to the number of walls as one-, two- or three-walled. If an increase in tooth mobility (≥ grade 1) was detected after surgery, the tooth was splinted. Figures 2 and 3 provide pertinent examples of the treatment procedure.

### Post-operative care

Starting with the day of the surgery, patients in the group treated with GTR/DBBM+AB (i.e., test) group were given 1-g amoxycillin (Ospamox, Sandoz GmbH, Kundl, Austria) twice daily for 7 days. Patients were instructed to rinse with 0.2% chlorhexidine digluconate solution for a period of 2 weeks (Eludril, Pierre Fabre Laboratories, Paris, France) twice a day. The sutures were removed 14 days after surgery. After 2 weeks, patients resumed brushing at the operated area using an ultrasoft post-surgical brush (Elgydium, Pierre Fabre Laboratories, Paris, France).

During the 12-month observation period, patients were recalled at 1, 2, 4 weeks, and 2, 3, 6, and 12 months postoperatively to detect any potential infection/suppuration and/or wound dehiscence during the early wound healing phase. Additionally, the level of oral hygiene was monitored and supragingival tooth cleaning was performed at the treated sites. At 3, 6, and 12 months, FMPS and FMBS scores were also recorded. Intraoral photographs were taken at every visit.

### Reentry procedure

Reentry procedure was performed at 1 year following regenerative surgery in those patients who have provided written informed consent (i.e., 23 out of 41 patients). Following local anesthesia, the mucoperiosteal flap was delicately elevated in order to measure the residual defect depth and width. Subsequently, the flap was repositioned and stabilized with single interrupted sutures. A 0.2% chlorhexidine digluconate solution (Eludril, Pierre Fabre Laboratories, Paris, France) was prescribed for the next 2 weeks. Sutures were removed at 7 days after reentry surgery.

### Statistical analysis

Data analysis was made with a commercially available software program (Statistica 13.1 software, Tulsa, USA). The study was designed for testing superiority. The statistical unit was the patient. The primary outcome variable was CAL gain from baseline to 12 months. For these calculations, only the same, at baseline the deepest site also exhibiting ≥ 6 mm, was included. The values were expressed as the mean and standard deviation. Comparisons within the groups were made with the Wilcoxon matched pairs signed-rank test while between the groups with the Mann-Withney rank-sum test. A *p* value of < 0.05 was considered as statistically significant.

Sample size was calculated a priori performing assumption a standard deviation of CAL change 1 mm and to detect a mean difference of 1 mm with a test power of 80% on 32 subjects. However, considering possible drop-outs, 41 patients were recruited and randomized to the study.

## Results

Forty-one patients completed the study (27 women, 14 men; mean age 41.78) (Table 1). Wound healing was generally uneventful in both groups. Minimal (up to 2 mm) membrane exposure was found in seven control and six test patients. No signs of suppuration, extensive dehiscence, or swelling were observed throughout the entire study period and none of the patients reported intense pain, fever, or discomfort. Two patients from the test group reported hypersensitivity of the treated teeth while one patient from the control group reported a cold sore confined to the corner of the mouth. All patients maintained a good level of oral hygiene throughout the entire study period, as evidenced through low FMPS and FMBS scores (i.e., < 20%).

**Table 1:** Sample’s age/gender distribution and characteristics of respective intrabony defects

	GTR/DBBM+AB (test)	GTR/DBBM (control)
*n*	21	20
Sex	13F, 8M	14F, 6M
Mean age	44.67 ± 9.76	38.75 ± 8.27
Tooth position		
Incisors	3	4
Canines	4	6
Premolars	10	5
Molars	4	5
Distribution and configuration of intrabony defects		
1-wall	5	6
2-wall	12	9
3-wall	4	5

No statistically significant differences in terms of PD, GR, CAL, and radiographic (RxD, RxW) parameters were found at baseline between the 2 groups. At 1 year, both groups demonstrated statistically significant improvements in terms of PD reduction and CAL gain. Compared with baseline, GR increased statistically significantly in the control group, but not in the test group. In both groups, RxD and RxW improved statistically significantly compared with baseline. Changes in clinical and radiological parameters are shown in Table 2. No statistically significant differences were found in any of the investigated parameters between the two groups after 1 year. The frequency distribution of CAL gain for both treatment groups and number of residual pockets (PD˃5 mm) is shown in Table 3. In the test group, 16 sites (76%) gained at least 3 mm of CAL. In the control group, no CAL gain occurred in two sites (10%), whereas at ten sites (50%), the CAL gain was 2 mm. A CAL gain of 3 mm or more was measured in eight defects (40%). In both groups, there were two residual pockets (PD ˃ 5 mm) left.

**Table 2:** Clinical recordings and radiographic measurements in test (GTR/DBBM+AB) and control (GTR/DBBM) group at baseline and 1 year post-op

	Baseline (mean ± SD)	12 months (mean ± SD)	*p*	Diff ± SD
PD (mm)				
Test	7.8 ± 1,5	4.0 ± 0.9	< 0.0001	3.8 ± 1.3
Control	7.4 ± 1,3	4.1 ± 1.3	< 0.001	3.3 ± 1.7
		*p* = 0.8		
GR (mm)				
Test	0.9 ± 1,2	1.0 ± 1.2	*p* = 0.29(NS)	− 0.2 ± 0.8
Control	1.2 ± 1,3	1.8 ± 1.6	< 0.05	− 0.6 ± 0.9
		*p* = 0.1		
CAL (mm)				
Test	8.7 ± 1,4	5.0 ± 1.7	< 0.0001	3.6 ± 1.6
Control	8.6 ± 1,9	5.9 ± 1.6	< 0.001	2.7 ± 1.6
		*p* = 0.07		
RxD (mm)				
Test	5.2 ± 2,1	2.0 ± 1.9	< 0.0001	3.2 ± 2.1
Control	4.8 ± 1,6	2.3 ± 1.2	< 0.001	2.5 ± 1.9
		*p* = 0.3		
RxW (mm)				
Test	2.8 ± 0,9	1.6 ± 1.0	< 0.001	1.3 ± 0.9
Control	3.1 ± 0.7	2.0 ± 1.2	< 0.001	1.1 ± 1.0
		*p* = 0.2

**Table 3:** Varying levels of CAL gain, bone gain, and number of residual pockets PD > 5 mm at 1 year after treatment

	GTR/DBBM+AB (test)	GTR/DBBM (control)
CAL gain < 2 mm	1/21	2/20
4 mm ≤ CAL gain ≥ 2 mm	15/21	15/20
CAL gain > 4 mm	5/21	3/20
INTRA DD gain < 2 mm	1/11	4/12
INTRA DD loss	0/11	0/12
residual pockets PD > 5 mm	2/21	2/20

Reentry surgery was performed in 23 out of the 41 patients (i.e., in 11 patients from the test group and in 12 patients from the control group, respectively). In both groups, a statistically significant reduction of INTRA DD and INTRA DW was measured compared with baseline, however without statistically significant differences between the groups (Table 4) .

**Table 4:** Clinical intraoperative recordings in the test (GTR/DBBM+AB) and control (GTR/DBBM) group

	Baseline (mean ± SD)	12 months (mean ± SD)	*p*	Diff± SD
Intra DD				
Test (*n* = 11)	5.5 ± 2.0	1.9 ± 0.9	< 0.01	3.7 ± 1.8
Control (*n* = 12)	5.0 ± 1.6	2.3 ± 1.1	< 0.01	2.7 ± 2.1
		*p* = 0.5		
Intra DW				
Test (*n* = 11)	3.7 ± 1.0	2.2 ± 1.3	< 0.05	1.5 ± 1.5
Control (*n* = 12)	3.8 ± 0.9	2.6 ± 1.3	< 0.05	1.1 ± 1.4
		*p* = 1.0		

## Discussion

The present study has evaluated the potential effects of a postoperative antibiotic regimen including the use of amoxicillin on the outcomes of regenerative periodontal surgery using the combination of DBBM and GTR. At 12 months following treatment, the results have failed to reveal statistically significant differences in any of the evaluated clinical parameters, despite some minor improvements that were favoring the additional use of systemic antibiotics. At 12 months following therapy, mean CAL gain measured 3.6 ± 1.6 mm in the test group with the corresponding value of 2.7 ± 1.6 mm in the control one. These results are in line with those of previous studies using a comparable regenerative approach and antibiotic protocol [22–24]. In those studies, the CAL gain measured 3.2 mm at 6 months [22], and 4.0 mm [23] and 3.9 mm [24], respectively. Interestingly, comparable results were also obtained in other studies where DBBM and GTR were used without postoperative administration of systemic antibiotics (i.e., 4.1 mm by Sculean et al. [25] and 3.7 mm by Iorio-Siciliano et al. [26]).

The rationale of using the combination of DBBM and GTR is based on the findings from histologic studies from animal models and human case reports/case series which have provided evidence for periodontal regeneration (i.e., formation of cementum, periodontal ligament, and bone) following this treatment modality [1, 27–29]. From a clinical point of view, it is important to emphasize that substantial evidence from randomized controlled clinical studies suggests that in intrabony defects, regenerative periodontal surgery with DBBM + GTR by means of collagen membranes may lead to improved clinical outcomes in terms of CAL gain and PD reduction compared with those achieved with open flap debridement alone [10] and corroborate the previously mentioned histologic findings [1, 27–29].

Thus, taken together, the available histological and clinical data suggest that the clinical improvements observed following regenerative surgery with DBBM and GTR may reflect, at least to a certain extent, periodontal regeneration.

In the present study, both treatments resulted in statistically significant radiographic fill of the intrabony defects. The mean RxD gain measured 3.2 ± 2.1 mm in the test group and 2.5 ± 1.9 mm in the control one. This compares well to the findings of other authors who have used a comparable regenerative approach [15]. In a study including a total of 120 intrabony defects treated in 10 different research centers, the radiographic resolution of the intrabony component measured 3.2 mm in the DBBM + GTR group [15]). However, when considering the value of an x-ray examination, one should bear in mind the possibility of some errors that may result from the difference in x-ray projection. Although in the present study the best possible attempts were made to standardize the x-rays by using a stent to allow a similar positioning of the film for all examination time points, it cannot be excluded that some minor differences between the x-ray projection occurred [7]. Therefore, the radiographic measurements should be interpreted with caution and be always considered in the light of the clinical outcomes.

In order to additionally verify the outcomes, a reentry procedure was performed in 11 out of 21 defects of the test group and in 12 out of 20 defects of the control group. The measurements revealed that in the test group, mean INTRA DD decreased from 5.5 ± 2.0 mm to 1.9 ± 0.9 mm (3.7 ± 1.8 mm INTRA DD gain), while the corresponding value changed from 5.0 ± 1.6 mm to 2.3 ± 1.1 mm (2.7 ± 2.1 mm INTRA DD gain) in the control group. None of the re-entered defects showed bone loss, i.e., 91% of test and 67% of control defects showed at least 2-mm fill of the intrabony component. Similar results following re-entry were also reported by Camargo et al. (2000) (i.e., 3.7 to 3.8 mm of defect fill as measured from an acrylic stent) [22]. When interpreting the value of re-entry procedures after regenerative periodontal surgery, it is important to be aware of the fact that such interventions are unable to provide evidence for periodontal regeneration (i.e., formation of cementum, periodontal ligament, and bone) and may thus only provide additional information to support the clinical and radiographic findings [1, 2].

The potential additional benefit of using systemic antibiotics in conjunction with regenerative surgery has been investigated in previous studies using as regenerative material an enamel matrix derivative (EMD) alone or combined with bone substitutes or membranes [13, 14, 30].

The rationale to select amoxicillin as antimicrobial agent was based on the fact that it covers a wide variety of gram-positive and gram-negative bacteria and is frequently used to minimize postoperative complications following third molar and implant surgeries [31, 32]. Since the aim of regenerative periodontal surgery is not to treat bacterial caused periodontal infection, but to reconstruct the defects left following nonsurgical anti-infective periodontal therapy, the postoperative administration of antibiotics intends to prevent potential postoperative complications related to the insertion of foreign materials such as grafts and membranes [1, 2].

Sculean et al. [13] have treated 34 patients with one deep intrabony defect by means of OFD and EMD followed by a postoperative protocol with or without systemic antibiotics. In that study, the antibiotic regimen consisted of a combination of 3 × 375 mg amoxicillin and 3 × 250 mg metronidazole daily for 7 days. At 1 year following therapy, both treatments resulted in statistically significant PD reduction and CAL gain (i.e., in the EMD + AB group, mean PD decreased from 9.1 ± 1.5 mm to 4.5 ± 1.1 mm (*p* < 0.0001) and mean CAL changed from 11.0 ± 1.6 mm to 7.5 ± 1.4 mm (*p* < 0.0001), while in the EMD group, mean PD decreased from 9.0 ± 1.7 mm to 4.3 ± 1.7 mm (*p* < 0.0001) and the CAL changed from 10.6 ± 1.6 mm to 7.3 ± 1.5 mm (*p* < 0.0001). No statistically significant differences were found in any of the investigated clinical parameters between the 2 groups [13].

Comparable results were also reported by Röllke et al. and Eickholz et al. [14, 30]. Following treatment of intrabony defects with either EMD, EMD + bone substitute or membrane. In that study the postoperative regimen consisted of either 200-mg doxycycline per day or placebo for a period of 7 days. The clinical measurements at 6, and 12 or 24 months after regenerative surgery failed to reveal statistically significant differences between the groups, thus questioning the clinical benefit of a postoperative administration of systemic antibiotics following regenerative periodontal surgery. Despite the fact that the biomaterials used in the mentioned studies have either used EMD alone or EMD and a bone grafting material without barrier membranes, the findings are in line with those obtained in the present study, thus questioning the routine administration of systemic antibiotics following regenerative periodontal surgery, provided that an optimal level of plaque control and maintenance care is ensured [13, 14, 30].

An important aspect that needs to be carefully discussed when interpreting the present results is the lack of placebo in the control group. Since no placebo was given, the used drug was not blinded and was given to the patients in the original package. Therefore, the patients were aware of the allocated treatment, which, obviously, is a shortcoming of the study. The lack of a placebo may therefore explain, at least partly, the tendency, though statistically not significant, for the improved clinical outcomes in the test group compared with the control one (i.e., 3.6 ± 1.6 mm CAL gain in the test vs. 2.7 ± 1.6 mm in the control group). On the other hand, the present results compare well to the studies mentioned previously where regenerative procedures were performed either with [14, 30] or without the use of a placebo [13].

In the present study, we have tried to minimize the possibility of bias by the fact that neither the examiner nor the surgeon was aware of the treatment allocation (i.e., test or control). All clinical measurements were made by the same blinded (i.e. the examiner was not aware of the allocated treatment groups) and calibrated examiner (ED), who was not the same as the surgeon. Additionally, the surgical interventions were scheduled in a way that the surgeon has only met the patient immediately before regenerative surgery and at the reentry surgery (in cases where re-entry was performed).

In conclusion, within their limits, the present results have failed to show added clinical improvement following the administration of systemic amoxicillin in conjunction with regenerative periodontal surgery using DBBM and GTR.
